# Magnetic Cell Labeling of Primary and Stem Cell-Derived Pig Hepatocytes for MRI-Based Cell Tracking of Hepatocyte Transplantation

**DOI:** 10.1371/journal.pone.0123282

**Published:** 2015-04-09

**Authors:** Dwayne R. Roach, Wesley M. Garrett, Glenn Welch, Thomas J. Caperna, Neil C. Talbot, Erik M. Shapiro

**Affiliations:** 1 Molecular and Cellular Imaging Laboratory, Department of Radiology, Michigan State University, East Lansing, Michigan, United States of America; 2 Animal Biosciences and Biotechnology Laboratory, Agricultural Research Service, Beltsville Agricultural Research Center, United States Department of Agriculture, Beltsville, Maryland, United States of America; University of Cagliari, ITALY

## Abstract

Pig hepatocytes are an important investigational tool for optimizing hepatocyte transplantation schemes in both allogeneic and xenogeneic transplant scenarios. MRI can be used to serially monitor the transplanted cells, but only if the hepatocytes can be labeled with a magnetic particle. In this work, we describe culture conditions for magnetic cell labeling of cells from two different pig hepatocyte cell sources; primary pig hepatocytes (ppHEP) and stem cell-derived hepatocytes (PICM-19FF). The magnetic particle is a micron-sized iron oxide particle (MPIO) that has been extensively studied for magnetic cell labeling for MRI-based cell tracking. ppHEP could endocytose MPIO with labeling percentages as high as 70%, achieving iron content as high as ~55 pg/cell, with >75% viability. PICM-19FF had labeling >97%, achieving iron content ~38 pg/cell, with viability >99%. Extensive morphological and functional assays indicated that magnetic cell labeling was benign to the cells. The results encourage the use of MRI-based cell tracking for the development and clinical use of hepatocyte transplantation methodologies. Further, these results generally highlight the importance of functional cell assays in the evaluation of contrast agent biocompatibility.

## Introduction

For many severe, progressive liver diseases, the only effective treatment is liver transplantation. Due to the shortage of available donor organs, liver transplantation is not available to all patients who might benefit. Alternatives to liver transplantation are an active area of research and include gene therapy and cell-based therapies, such as cell transplantation and artificial liver bio-devices.

Cell-based therapies, such as hepatocyte transplantation, have not yet become a sustainable treatment for patients with acute liver failure and chronic liver disease [1–4]. Ideally, fresh human hepatocytes from a healthy donor would serve as the cell source for such a therapy. Yet, as with intact livers, supply is outstripped by demand. Furthermore, there are currently no robust protocols for expanding hepatocytes in culture, and the functional phenotype of primary human hepatocytes is challenging to maintain *in vitro* [5,6].

**Fig 1:**
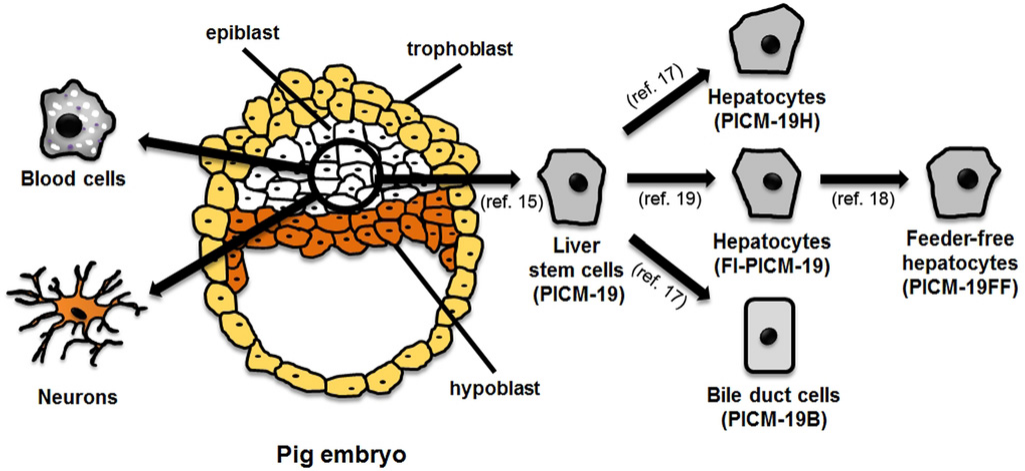
Schematic of the isolation of the hepatocyte-like cell line, PICM-19FF, from pig embryo epiblast cells.

Strategies to overcome these limitations include cells of xenogeneic origin [7], such as from swine [8]. While primary human hepatocytes are extremely limited, the supply of primary pig hepatocytes (ppHEP) is essentially unlimited, and, therefore, ppHEP could serve a role in treating liver diseases in humans. An alternative to primary liver cells are stem cell (SC) or induced pluripotent stem cell (iPSC)-derived hepatocytes that could potentially offer endless quantities of hepatocytes for cell replacement therapy [9–12]. In the case of pig hepatocytes derived from stem cells, Talbot et al. have described a bipotent liver stem cell line derived from a pig embryo’s pluripotent epiblast tissue (Fig 1) [13–15]. The cell line, designated PICM-19, displayed the property of spontaneous differentiation into the two parenchymal cell types that comprise the liver, hepatocytes and cholangiocytes (bile duct epithelial cells) and proliferate indefinitely, i.e, are an immortal cell line. The PICM-19 cell line, however, was dependent on co-culture with mouse fibroblast feeder-cells for its growth and for the maintenance of its bipotent differentiation potential [14,16,17]. To remove the complication of the presence of feeder-cells in the culture, a feeder-cell-independent subpopulation of the PICM-19 cell line, PICM-19FF, was isolated from the parental cells [18,19]. The PICM-19FF cells retain a hepatocyte phenotype and the ability of unlimited self-renewal without the need for direct contact with feeder cells, making them appropriate for cell transplantation therapy studies.

Regardless of the source of cells, research and clinical applications of cell transplantation therapies are limited by the inability to effectively track the fate of cells once they have been infused into the patient [20,21]. Post-transplant evaluations in patients have typically relied upon measurement of enzymatic activities or soluble factors, which do not give insight into the migration or localization of the transplanted cells. Further, if the cell transplant fails, these factors will not be present, the late determination of which may doom the patient. Histological analysis of tissues obtained by biopsies provide evidence of localization and engraftment of transplanted cells but entail an intrinsic risk to the patient and do not permit serial monitoring [20,21]. The capability to detect and measure the extent of hepatocyte transplant would be paradigm shifting as it would enable physicians to consider additional hepatocyte transplantation regimens or second line treatments if hepatocyte transplantation fails. From a research perspective, it would allow development of improved transplantation strategies in large animals where whole organ histology is more difficult than in rodents.

As such, noninvasive imaging techniques are being explored to address the questions of transplanted cell migration, localization, viability and interaction with native hepatocytes in the liver. For example, Chouhan, et al, labeled human and rat hepatocytes with ^99m^Tc-GSA, a clinical scintigraphic agent which is specifically taken up by the hepatocyte asialoglycoprotein receptor [22]. This will allow the use of SPECT for determining hepatocyte transplant. Hickey, et al, also demonstrated the utility of SPECT imaging, instead using a reporter gene paradigm [23]. Hepatocytes were engineered to express the sodium iodide symporter, which transports radioactive iodine and ^99m^Tc- sodium pertechnate. After transplant, the delivery of these two radioactive materials allows the detection of transplanted hepatocytes.

MRI-based cell tracking has proven useful for visualizing the location of transplanted cells, *in vivo*. In short, donor cells are labeled with magnetic nano- or micro-particles which enable their detection as areas of dark contrast in recipient organs following transplantation [24]. An important challenge in MRI-based cell tracking is to quantify the number of cells being detected. Various methods, including relaxometry [25,26] and susceptibility field mapping [27], all suffer the same drawback, that is, it is impossible to discriminate between a pixel containing 100 cells labeled with 10 particles each and a pixel containing 10 cells labeled with 100 particles each. This is magnified by the degradation of the contrast agent over time [28], which further influences their MRI properties, complicating the data, especially in longitudinal studies. A solution to this problem is enumeration of punctate hypointensities in the MRI, i.e., counting dark spots, where each dark spot is generated by a single cell.

*In vivo* detection of single cells by MRI has been accomplished. For example, Shapiro et al. demonstrated this principle with transplanted hepatocytes in mouse liver [29]. Heyn et al. demonstrated *in vivo* detection of single cells by MRI in a model of tumor cell invasion into the brain, with excellent matching histology [30]. Wu et al. have detected single cells *in vivo* by MRI in models of organ rejection following transplant [31]. Common to all of these studies was the use of micron-sized iron oxide particles (MPIO) as the labeling agent. MPIO are commercially available (Bangs Laboratories, Fisher, IN) and are composed of an iron oxide core (>45% magnetite by weight) and coated with an inert polymer matrix, embedded with fluorescent dye [32–36]. The inert matrix potentially limits the clinical application of MPIOs, as particles do not degrade *in vivo* [37]. Biodegradable versions of MPIOs constructed from PLGA are emerging as well [38,39].

Appropriate preclinical animal models, along with MRI techniques, would allow for the development and refinement of cell transplantation methodology prior to advancing to clinical trials. Swine models of liver disease are considered a reliable model for liver failure because the pig’s liver has a similar anatomy, physiology and size compared with the human liver [40]. This is particularly true for modeling newborn liver diseases caused by inborn metabolic disorders of a genetic basis. This study evaluated the effects of 0.86 μm MPIO magnetic labeling of two cultures of pig liver cells, ppHEP and PICM-19FF pig liver stem cells, to establish their suitability for MRI-based cell tracking of transplantation. Since hepatocytes are highly metabolic, notable for a multitude of functions including energy metabolism, bile production, and detoxification, the study probed whether MPIO-labeled ppHEP and PICM-19FF cells maintained their cellular and metabolic functions. It was demonstrated by a series of hepatocyte-specific *in vitro* assays that, in fact, they do. These results pave the way to study the *in vivo* behavior of transplanted hepatocytes, which is an essential preclinical step in the development of human regenerative medicine therapies.

## Materials and Methods

### Cell cultivation and reagents

Unless otherwise stated, all cell culture reagents were purchased from Hyclone (Logan UT), and all chemical reagents were purchased from Sigma-Aldrich (St Louis, MO). Cell culture reagents include Dulbecco's phosphate buffered saline (DPBS) without Ca^++^ and Mg^++^.

PICM-19FF cells were cultured as previously described [19]. Briefly, cells were seeded at 1 x 10^6^ per T12.5 tissue culture flask (Becton-Dickinson Labware, Franklin Lakes, NJ) pre-coated with polymerized collagen (PureCol, Advanced Matrix, Inc., San Diego, CA) diluted 1:1 with high glucose Dulbecco’s Modified Eagles Medium (DMEM) supplemented with 10% fetal bovine serum (FBS; 10% DMEM) for a final concentration of approximately 1.5 mg collagen/mL. Twenty-four hours after seeding, the cells were overlaid with Matrigel (BD Biosciences, Bedford, MA) diluted 1:50 in a 1:1 mixture of DMEM (low glucose) and Medium 199, supplemented with 10% FBS, 0.1% 2-mercaptoethanol and nucleosides (10% DMEM/199) that had been conditioned on feeder-layers of irradiated Sandoz inbred strain, thioguanine- and ouabain-resistance (STO) mouse fibroblasts (CRL 1503, American Type Culture Collection, Rockville, MD). The cultures were fed daily with fresh STO conditioned 10% DMEM/199 thereafter.

ppHEP were isolated from crossbred female suckling piglets as previously described [41] and modified as described below. Care and treatment of all pigs in this study were approved by the Institutional Animal Care and Use Committee of the U.S. Department of Agriculture, Beltsville Area Animal Care and Use Committee (BAACUC) Protocol# 12–011. Piglets were anesthetized by intramuscular injection of sodium pentobarbital (100 mg/kg). Livers from 7–24 d pigs were excised intact and the entire left lateral lobe removed and perfused with 500 mL of 10 mM HEPES-buffered saline (HBS) containing 0.5 mM EGTA, followed by an additional 500 mL HBS. The liver matrix was digested by recirculating 121 units/mL collagenase (Type 2, Worthington, Lakewood, NJ) in a mixture of HBS containing 100 mM HEPES and Medium 199 (2:1) amended with CaCl_2_, glucose, bovine serum albumin (BSA) and bovine insulin through the lobe for ~30 min [41]. The lobe’s tissue mass was physically teased apart and washed 6X with Medium 199 with Hanks’ salts with 10 mM HEPES using low speed centrifugations (50 x g). Cells were seeded at 3 x 10^6^ per T12.5 flask coated with monomeric bovine skin collagen (Life Technologies, Grand Island, NY) in William’s E medium supplemented with 0.1 mM β-mercaptoethanol, 10 mM HEPES, 10 nM Na_2_SeO_3_, 2 mM glutamine, insulin, transferrin, antibiotics, and 10% FBS. After 3 h, the attached ppHEP were washed 2X with HBS, and fed William’s E supplemented with 5% FBS (5% Williams E) for 21 h. Macrophages were eliminated from the ppHEP cultures by exposing them to 10 ng/mL ricin-A in serum-free DMEM for 1 h, washing 4X in 10% DMEM, and culturing the ppHEP with 5% William’s E for 23 h. Thereafter, ppHEP were maintained in serum-free William’s E with 1 mM L-carnitine, 0.01% dimethylsulphoxide (DMSO), 0.1% BSA, 10 nM dexamethasone and 1 ng/mL of bovine insulin. All cell cultures were routinely maintained at 37°C and in a 5% CO_2_ atmosphere.

### MPIO labeling

Polystyrene-coated 0.86 μm MPIO containing fluorescent Dragon Green dye (480 nm excitation, 520 nm emission) (Bangs Laboratories, Fishers, IN) were used for cell labeling. Cells were incubated with various concentrations of MPIO in 2.5 mL of STO conditioned 10% DMEM/199 medium for 24 h and washed 3X with DPBS to remove excess MPIO. PICM-19FF cells were coated with Matrigel after the addition of MPIO since it would otherwise block the uptake of the MPIO by the cells. MPIO uptake was confirmed by phase-contrast, fluorescent, and transmission electron microscopy (TEM). Labeling efficiency was calculated by flow cytometry (FACSVerse; BD Biosciences, San Jose, CA) on cells dissociated with Accutase (Sigma-Aldrich) and resuspended in HBS with 0.2% BSA. Cell suspensions were analyzed using 488 nm excitation from a 20 mW solid state laser and its associated fluorescence detection filters, a 527/32 bandpass for cells labeled with green fluorescent MPIOs and a 700/54 bandpass for propidium iodide (PI). Ten thousand cells were selected by their light scatter to exclude small debris. Analysis regions were set to determine the percent of viable cells (PI negative) that were positive for MPIO using the instrument’s FACSuite software. Iron content was measured by inductively coupled plasma optical emission spectroscopy (ICP) after complete degradation of cells and internalized particles in nitric acid.

### Analysis of secreted proteins

PICM-19FF cells were washed 4X with serum-free, protein-free DMEM/Medium 199 medium and incubated in 2 mL of this medium for 72 h to prepare serum-free conditioned medium (SFCM). The SFCM was centrifuged at 500 x g to remove cell debris and concentrated approximately 14-fold using Vivaspin concentrator units (Sartorius Stedim Biotech, Bohemia, NY). Two-dimensional gel electrophoresis of the SFCM and Maldi-TOF/TOF mass spectroscopy protein identification was performed as previously described [42,43]. Unknown proteins were identified from TOF/TOF spectra using the Mascot search engine (Matrix Science, London, UK; version 2.3.02). A species specific FASTA formatted database was constructed for Mascot searching by downloading the protein reference sequence subset (38,411 sequences as of 04/09/14) from the National Center for Biotechnology Information non-redundant protein database using the taxonomy filter, *Sus scrofa*. The FASTA formatted sequences of common human keratin contaminants were appended to the database for a total of 38,418 sequences. The following parameters were used for TOF/TOF database searches: monoisotopic mass, parent ion tolerance of 50 ppm, fragment ion tolerance of 0.5 Da, peptide charge state set to 1+, trypsin as digesting enzyme with 1 missed cleavage allowed and variable modification of oxidation of methionine, N-terminal pyroglutamic acid from glutamic acid or glutamine. Scaffold (version Scaffold_4.3.0, Proteome Software Inc., Portland, OR) was used to validate TOF/TOF based peptide and protein identifications. Protein identifications were accepted if they could be established at greater than 95.0% probability and contained at least 2 identified peptides.

### γ-Glutamyl transpeptidase (GGT) histochemical staining

In situ histochemical staining of GGT activity was performed as described by Reutenberg et. al [44]. Confluent PICM-19FF cell monolayers were fixed with ice-cold methanol for 2 min and exposed to filtered substrate solution containing γ-Glutamyl-4-methoxy-2-naphthylamide, DMSO, glycylglycine and Fast blue BBN (diazotized 4’-amino-2’, 5’-diethoxybenzanilide) in Tris-buffered saline (pH 7.4) for 10 min at 37°C. Cells were rinsed with 0.85% saline, incubated with 0.1 M cupric sulfate for 2 min, rinsed in saline and photographed in fresh saline.

### Cytochrome P450 activity and urea production

Cytochrome P450 induction and urea production analyses were performed on cultures as previously described [45]. Briefly, ethoxyresorufin-o-deethylase (EROD) activity was used to measure microsomal hydrolysis of ethoxyresorufin to resorufin by the cytochrome P-450 1A (CYP 450 1A) after a 24 h exposure to 5 μM 3-methylcholanthrene (3MC). After exposure, cells were washed 2X with DPBS and maintained in 2.5 mL Medium 199 with Hanks’ salts (without L-glutamine), 8 μM 7-ethoxyresorufin, 10 μM dicumerol, and BSA for 30 min. Medium was collected and the concentration of the fluorescent product, resorufin, was obtained in the presence and absence of β-glucuronidase/arylsulfatase (Roche Applied Sciences, Mannhein, Germany) at 530/590 nm. To assess urea production, all cells were preincubated in medium containing 100 ng/ml porcine glucagon and 2 mM glutamine for 24 hr. Cultures were washed with warm HBS and replaced with glutamine and serum-free Williams E medium containing 100 ng/ml glucagon without (basal) or with 2 mM NH4Cl for 24 hr. The concentration of urea was determined colorimetrically based on a diacetyl monoxime reaction assay at 540 nm [46] as modified by WHO (http://apps.searo.who.int/PDS_DOCS/B0218.pdf) and compared to a urea nitrogen standard curve (Standard 535, Sigma-Aldrich). At the termination of both assays, cells were washed with HBS, scraped and total protein was determined by Lowry assay following precipitation in 7% TCA and 0.07% Triton-X 100, using BSA to prepare a standard curve.

### Statistical analysis

Quantitative data were analyzed by GLM ANOVA (NCSS 2007, Kaysville, UT) using standard factorial models. Values of P<0.05 were considered significant among treatment groups and no interactions were observed among any treatment variables. Paired t-tests were performed to directly compare control and MPIO-treated cells.

## Results

### MPIO uptake and cytotoxicity

**Fig 2:**
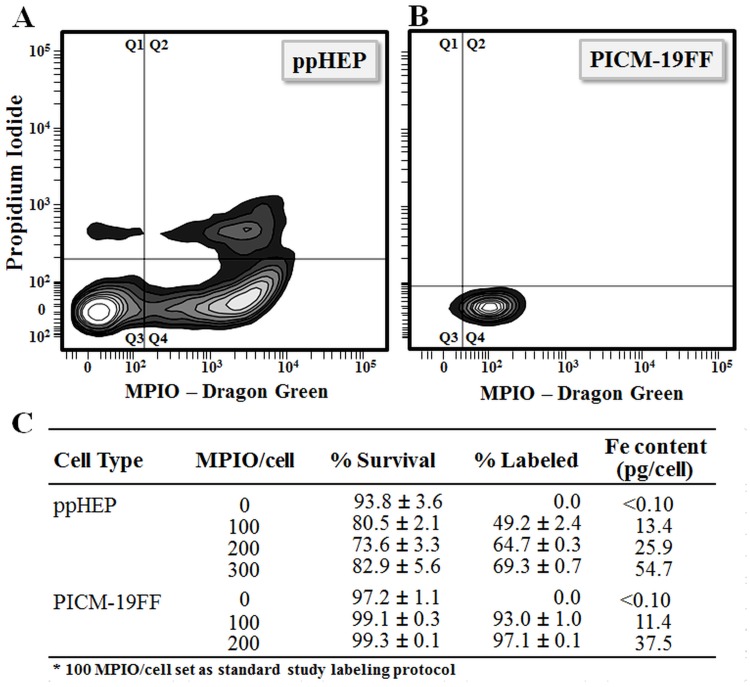
Cell survival and MPIO-labeling efficiency identified by flow cytometry in ppHEP and PICM-19FF cells. A-B) Representative example of flow cytometry of isolated ppHEP, or PICM-19FF cells, post incubation with 100 MPIO/cell. (Q1) unlabeled-dead cells (PI stained) (Q2) green MPIO labeled-dead cells (Q3) unlabeled-live cells (Q4) green MPIO labeled-live cells. C) Quantitation of flow cytometry (n = 3) and iron content (n = 1). For ppHEP, differences in % Survival for 100, 200 and 300 MPIO/cell were each statistically significant, p < 0.05, only compared to 0 MPIO/cell but not significant among themselves, while differences in % Labeled were all statistically significant from each other. For PICM-19FF, differences in % Labeled were all statistically significant while differences in % Survival were not.

Dose—and time–dependent experiments showed that uptake of 0.86 μm MPIO occurred spontaneously in both ppHEP and PICM19-FF cells. The percent of cells labeled with MPIOs was quantified using flow cytometry and total internalized iron was quantified using ICP. The number of labeled cells using a dose of 100 MPIO/cell was ~2-fold greater for PICM-19FF over ppHEP, averaging 93.0% and 49.2%, respectfully (Fig 2). Doubling the MPIO/cell dosage to 200 MPIO/cell increased the number of labeled PICM-19FF by ~4% and ~16% in ppHEP. Tripling the dosage to 300 MPIO of ppHEP further improved labeling to ~69%. Cell death calculated by FACS analysis of PI-stained cells, was negligible (~1%) in MPIO-labeled PICM-19FF cultures and ranged from 17–26% in ppHEP, but the relationship between MPIO load per cell and death rate was not apparent. Increases in internalized iron content followed both the increase in dosage of MPIO used and the % labeling, achieving iron contents ranging from 11.4–54.7 pg iron/cell. Labeling culture flasks with 100 MPIO/cell was deemed adequate for MRI cell detection [29], therefore this concentration was used throughout the rest of the study.

**Fig 3:**
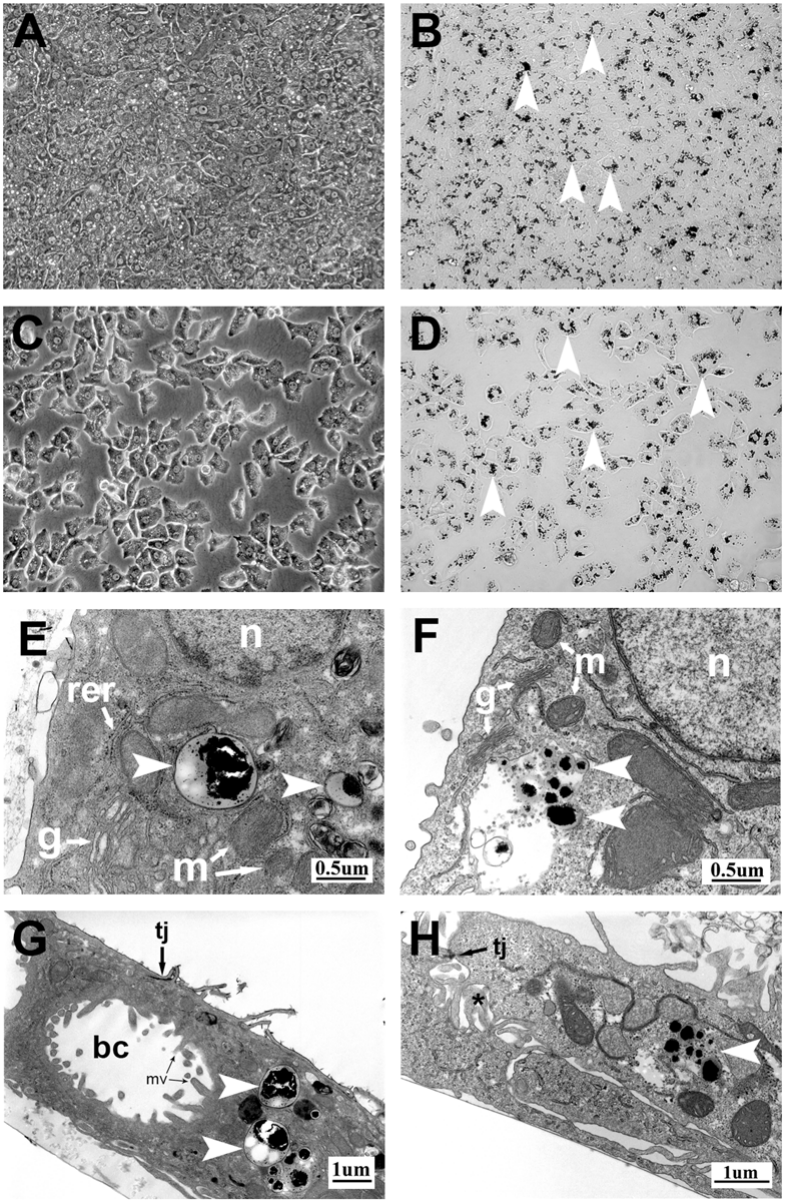
Microscopy of ppHEP and PICM-19FF cells labeled with 0.86 μm MPIO at 100 MPIO/cell after 24 h. Panels A and C are phase-contrast images of labeled ppHEP and PICM-19FF cells, respectively (200x). Panels B and D are corresponding bright-field images showing peri-nuclear MPIO particles sequestered within the cells (arrowheads). Transmission electron micrographs of MPIO-loaded PICM-19FF (Panels E and G) and ppHEP cells (Panels F and H) with arrowheads denoting the intracellular MPIO. Note that the morphology of the cells, their cellular junctions, their intimate cell-to-cell interdigitions (*), and their internal organelles appear normal [16, 19]. bc = biliary canaliculus, g = Golgi apparatus, n = nucleus, m = mitochondrion, mv = microvillus, rer = rough endoplasmic reticulum, tj = tight junction-like structure.

The morphology of PICM-19FF and ppHEP cells, and their cell-to-cell unions, appeared to be unaffected by the internalized MPIO (Fig 3A and 3C). Light microscopy showed the MPIO were found throughout the cells’ cytoplasm, although they were often perinuclear in distribution (Fig 3B and 3D). MPIO-labeling in either cell type did not appear to cause cell necrosis or apoptosis, evidenced by the cells’ sharply defined cell membranes and the maintenance of their cuboidal shape with distinct, centrally located nuclei. Ultrastructural features of the MPIO-labeled PICM-19FF cells were similar to non-labeled cells (Fig 3E and 3F) and the cells’ organelles looked typical of healthy cells showing no obvious deleterious changes [16, 19]. The internalized MPIO were often membrane bound and were found in the cytoplasm either freely or within digestive vacuoles (Fig 3E–3H). Intimate cell-to-cell associations with cytoplasmic interdigitations were maintained by the cells, and junctional apparati typical of polarized epithelial cells were found between cells and delineated their biliary canaliculi, when they occurred (Fig 3G and 3H). As is typical, the biliary canaliculi had numerous microvilli that protruded into the canalicular space (Fig 3G and 3H).

### Bile canaliculi functions of MPIO-labeled PICM-19FF

**Fig 4:**
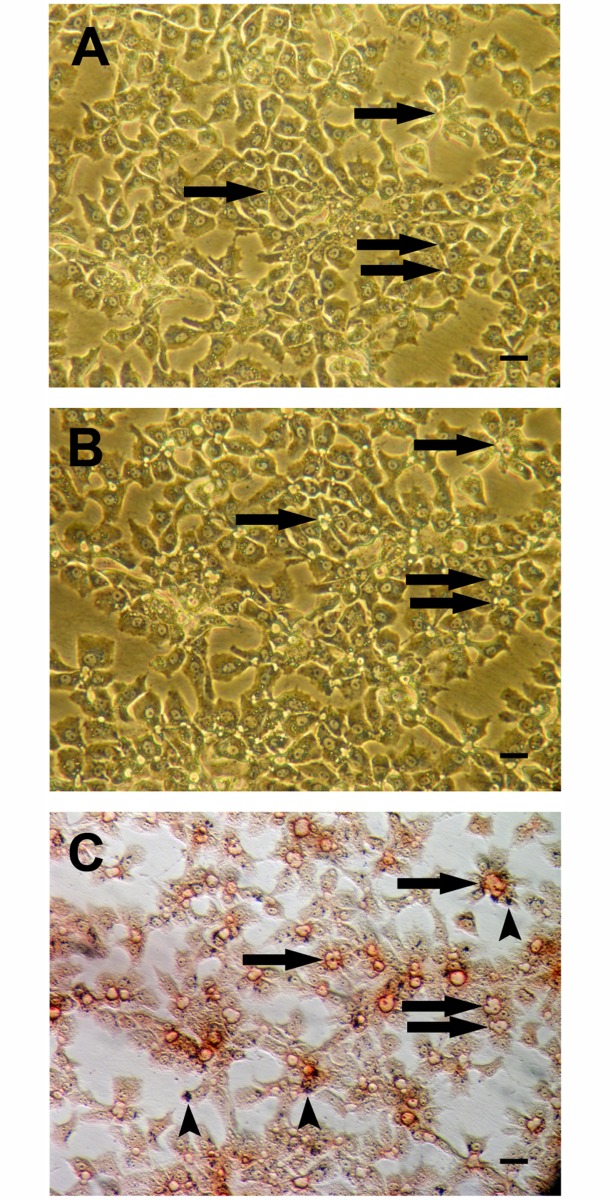
MPIO-loaded PICM-19FF cells stained for GGT activity 20 min after the addition of glucagon (100 ng/mL final) to the culture medium. Panels A and B are phase-contrast images of the same area before and after, respectively, the addition of glucagon (200x). C) The same area photographed with Hoffman modulation after histochemical staining for GGT activity (200x). Note the intense GGT histochemical staining at the apical cell membranes comprising the biliary canaliculi (arrows). Arrowheads indicate MPIO iron particles. Scale bar = 16 μm.

The effect of MPIO labeling on intracellular signal transduction by cyclic adenosine monophosphate (cAMP) was tested by glucagon treatment. Monolayers of MPIO-labeled PICM-19FF cells displayed typical enlargement of biliary canaliculi in response to glucagon (100 ng/mL) within 10–15 min (compare Fig 4A and 4B), presumably as a result of transcellular fluid transport into the canalicular space [14].

Gamma-glutamyl transpeptidase (GGT) is a membrane-bound glycoprotein present primarily in cells that have a secretory or absorptive function, such as the parenchymal cells of the liver and their biliary canalicular structures. Histochemical staining showed the expected GGT activity in MPIO-labeled PICM-19FF cells located in the apical cell membranes of the bile canaliculi formed between adjacent cells (Fig 4C) [18]. MPIO-labeled PICM-19FF cells not associated with one another, that is, without canaliculi, were negative for GGT activity.

### Serum protein production by MPIO-labeled PICM-19FF

**Fig 5:**
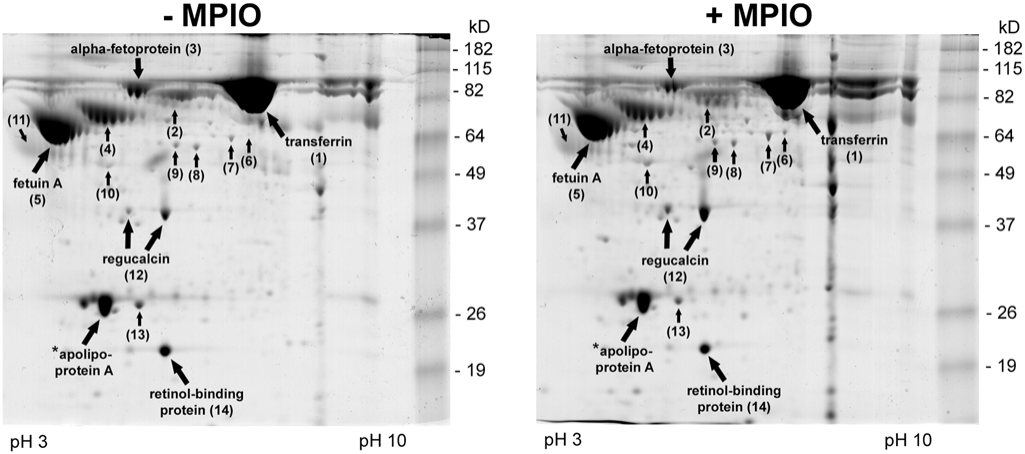
Two-dimensional electrophoretic polyacrylamide gels of conditioned serum-free medium from MPIO-labeled and unlabeled PICM-19FF. Proteins are indicated by spot number and name as identified by MALDI-TOF-TOF mass spectroscopy (see Table 1). *Previously identified; see Talbot et al. [43].

The SFCM from MPIO-labeled and unlabeled PICM-19FF culture was analyzed by 2D-gel electrophoresis and mass spectroscopy. The 2D-gel protein profile of the SFCM showed a typical array of serum proteins was produced by the PICM-19FF cells. The protein profiles of the MPIO-labeled and unlabeled cells were nearly indistinguishable (Fig 5). Of the 17 identified proteins from the 14 gel spots selected for analysis, 12 were secreted serum proteins (transferrin, serum albumin, alpha-fetoprotein (AFP), alpha-1-antitrypsin, alpha-2-HS-glycoprotein (fetuin-A), alpha-1-acid glycoprotein, fibrinogen gamma chain, fetuin-B, transthyretin, vitronectin, apolipoprotein E, and retinol-binding protein 4) and the remaining five were determined to be intracellular proteins (alpha-enolase, actin, regucalcin, isopentenyl-diphosphate Delta-isomerase 1, and 4-hydroxyphenylpyruvate dioxygenase) (Fig 5; Table 1). To ensure that the serum proteins were true secretion products from the PICM-19FF cells, and not carryover products from FBS, the TOF/TOF spectra were initially searched against both bovine and porcine reference sequences. Serum albumin was the only protein that had peptides matched to bovine as well as porcine sequences. In total, 4 peptides could be matched uniquely to bovine serum albumin, 2 peptides were found to be in common between bovine and porcine albumins, while 14 peptides were found to be unique to only porcine albumin (data not shown).

**Table 1:** Maldi-TOF/TOF mass spectrometry analysis.

Spot number	Protein name and species identification	Protein accession numbers	Protein MW (Da)	Protein identification probability	Exclusive unique peptide count	Percentage sequence coverage
1	serotransferrin precursor [Sus scrofa]	gi|347582654	78,919.40	100.00%	9	15.50%
2	PREDICTED: serum albumin isoform X1 [Sus scrofa]	gi|545845360	69,792.50	100.00%	13	20.10%
3	alpha-fetoprotein precursor [Sus scrofa]	gi|47523700	68,625.00	100.00%	11	16.90%
3	PREDICTED: serum albumin isoform X1 [Sus scrofa]	gi|545845360	69,792.50	98.70%	2	4.21%
4	alpha-1-antitrypsin precursor [Sus scrofa]	gi|47523844	47,195.60	100.00%	8	25.70%
5	PREDICTED: fetuin A [Sus scrofa]	gi|545865183	38,787.30	99.90%	2	6.58%
6	PREDICTED: alpha-enolase isoform X1 [Sus scrofa]	gi|545833443	38,083.50	100.00%	5	20.10%
7	PREDICTED: alpha-enolase isoform X1 [Sus scrofa]	gi|545833443	38,083.50	100.00%	7	27.10%
8	PREDICTED: fibrinogen gamma chain-like [Sus scrofa]	gi|335308461	49,620.50	100.00%	7	21.50%
9	PREDICTED: fibrinogen gamma chain-like [Sus scrofa]	gi|335308461	49,620.50	100.00%	6	16.40%
9	transthyretin precursor [Sus scrofa]	gi|47523508	16,081.70	100.00%	4	42.00%
9	PREDICTED: fetuin-B-like [Sus scrofa]	gi|311269755	41,223.90	99.30%	2	6.37%
10	PREDICTED: actin, cytoplasmic 1 [Sus scrofa]	gi|311250866	41,793.90	100.00%	9	29.90%
10	4-hydroxyphenylpyruvate dioxygenase [Sus scrofa]	gi|47523532	45,064.50	100.00%	3	10.70%
11	PREDICTED: alpha-1-acid glycoprotein-like [Sus scrofa]	gi|545804155	23,046.10	100.00%	4	20.20%
11	PREDICTED: vitronectin isoform X1 [Sus scrofa]	gi|545892823	52,572.60	99.90%	2	7.64%
12	regucalcin [Sus scrofa]	gi|116175265	33,117.80	100.00%	7	34.40%
12	PREDICTED: isopentenyl-diphosphate Delta-isomerase 1 isoform X1 [Sus scrofa]	gi|545854115	26,536.00	100.00%	2	11.10%
13	apolipoprotein E precursor [Sus scrofa]	gi|47523674	36,598.70	100.00%	11	35.30%
14	retinol-binding protein 4 precursor [Sus scrofa]	gi|47522930	23,039.10	100.00%	4	16.40%

### P450 and urea production by MPIO-labeled ppHEP and PICM-19FF

**Fig 6:**
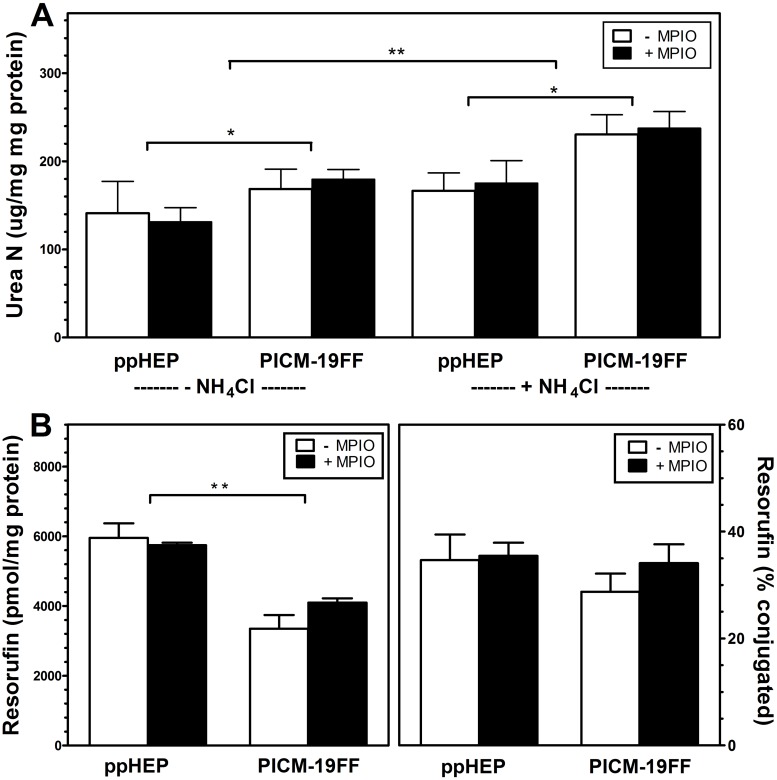
Urea production and cytochrome P450 activity of MPIO-labeled and unlabeled cells. A) Urea production with and without the addition of 5 μmol NH_4_Cl to the culture medium. B) P450 EROD activity after a 48-h 3-methylcholanthrine (5 μM) induction. Total fluorescent activity is with the addition of β-glucuronidase/arylsulfatase so as to detect total (left) and conjugated (right) EROD activity products. (n = 3, bars represent SEM) **P<0.01, ***P<0.001

Hepatocytes normally convert circulating ammonia into urea. PICM-19FF cells and ppHEP both synthesized and secreted significant basal amounts of urea during culture without added ammonia (Fig 6A). These basal amounts were not altered by MPIO labeling in either cell type. The addition of 2 mM NH_4_Cl (70 μg N/flask) increased average urea N by 28% in PICM-19FF cells and 20% in ppHEP (ANOVA; P<0.01). The conversions of added ammonia N to urea N ranged between 62% and 81% in 24 h and was not different between the two cell types (P>0.05), and were unaffected by MPIO labeling in either cell type.

The 7-ethoxyresorufin-O-deethylase (EROD) activity monitors the induction of the xenobiotic-metabolizing enzyme CYP1A1 in response to exposure to xenobiotics; in this case 3MC (Fig 6B). Total mean EROD activities were not significantly different (t-test; P>0.05) between MPIO-labeled (4.0 x 10^3^ ± 125 pmol/30 min/mg of protein; n = 3) and unlabeled (3.3 x 10^3^ ± 396 pmol/30 min/mg of protein; n = 3) PICM-19FF cells. Similarly, total mean EROD activities were not significantly different (t-test; P>0.05) between MPIO-labeled (5.7 x 10^3^ ± 73 pmol/30 min/mg of protein; n = 3) and unlabeled (5.9 x 10^3^ ± 423 pmol/30 min/mg of protein; n = 3) ppHEP. Total mean EROD activity from ppHEP was 57% greater than PICM19-FF cells (ANOVA; P<0.001), while the observed levels of conjugated resorufin (~35% of total resorufin) in media were similar among control and MPIO labeled ppHEP and PICM-19FF cells (ANOVA; P>0.05). The amount of the total secreted resorufin that can only be measured following incubation with arylsulfatase/β glucuronidase (i.e., percent conjugation) is an indication of the phase II detoxification enzyme capacity within the cells.

## Discussion

Hepatocyte transplantation requires further optimization for complete clinical benefit and both primary and stem cell-derived hepatocytes are worthy of investigation. This study analyzed the effects of MPIO labeling on freshly isolated and cultured pig hepatocytes, ppHEP, and a stem cell derived hepatocyte cell line, PICM-19FF. For both cell types, cellular labeling occurred in an MPIO dose-dependent manner through simple incubation with the cells in culture. Using a series of *in vitro* assessments that evaluated hepatic morphology, serum-protein production, responsiveness to cAMP signal transduction, inducible xenobiotic-metabolizing P450 activity and ammonia-induced urea production, we demonstrated that neither cell types’ morphology nor hepatic functions were impacted or impaired by intracellular uptake of MPIO. These results provide evidence that MPIO labeling is generally nontoxic to the cells and suggests that both magnetically labeled ppHEP and PICM-19FF cells may be useful for MRI-based cell tracking studies of cell engraftment in liver failure models.

Liver cell transplantation in humans has proved to be feasible and safe, and the majority of the cases demonstrated clinical improvements over time. However, it has not yet achieved sustainable benefits for patients due to the poor repopulating capacity of engrafted cells [11,47,48]. Possible reasons for the unsustainable long-term outcome of transplanted cells may be insufficient cell translocation through the endothelial cell barrier, insufficient cell engraftment into the liver plates and destruction of the engrafted cells by the immune system. Thus, an optimization of cell therapies in humans is needed and this could be performed in swine models.

We have selected a stem cell derived pig hepatocyte, PICM-19FF, that has been shown to have several key hepatocyte morphological and functional properties including, but not limited to, serum-protein production, P450 activities, ammonia clearance and urea production [for review see [13]]. Here, the PICM-19FF cell line was found to behave essentially the same as freshly isolated ppHEP from 7–21 d pigs, both before and after magnetic particle labeling and as demonstrated in a series of hepatocyte specific assays. The only evident difference for PICM-19FF cells was their greater affinity for MPIO labeling, by reasons unknown. The PICM-19FF cell line might have considerable potential in transplantation therapies, with advantages over other cell sources such as its; (i) infinite propagation in culture, (ii) growth independent of contact with mouse derived feeder cells (iii) normal growth rate, hepatocyte differentiation and function, (iv) simplified culture composition, i.e., single, consistent and defined cell type, and (v) ability to be selected for or genetically engineered for potential enhancement of cell functions and decreased immunogenicity.

There is precedent for the use of pig hepatocytes in both allogeneic cell transplantation animal models (pig-to-pig) and xenotransplantation models, e.g., pig to primate. Pig hepatocytes have been transplanted into the peritoneal cavity of pigs following cell encapsulation into polymer minitubes, and they exhibited hepatic structural and functional features for up to 21 d, without the need for immunosuppression [49]. In a pig model of acute liver failure induced by D-galactosamine poisoning, transplantation of pig hepatocytes prolonged the survival of the D-gal-treated pigs [50]. Direct intrasplenic injection of pig hepatocytes has been shown to correct the physiologic abnormalities associated with decompensated liver disease in pigs [51].

In addition to development of transplantation strategies in large animals, xenotransplantation of pig hepatocytes to humans could offer an immediate solution to supply constraints or to bridge the time until a suitable human donor organ is available [8,52]. Pig cell xenotransplantation has been performed with some success in animal models. In non-human primates, Nagata et al. [8] reported the longest survival of pig hepatocyte transplants—by intrasplenic infusion of cells—with hepatocyte function lasting for more than 253 days. There was no discernible rejection of the pig hepatocytes as evidenced by a lack of anti-pig cell antibodies in the primate serum [8]. Experimental results obtained with xenotransplantation of other pig cell types to humans such as pig-islets and neuronal-cells, have similarly been encouraging [for review see [7]]. Xenotransplantation of pig hepatocytes should be further explored because they are readily available, and, with the advancements in the genetic modification of pigs to humanize their organs and cells, it is possible that improvements to pig hepatocyte functionality and immune tolerance are attainable [7].

Further development of hepatocyte transplantation strategies could benefit from dynamic and serial monitoring of cell transplants, particularly in large animals. Only a few studies have investigated the use of MRI monitoring of MPIO-labeled liver cell transplantation in a large animal model [53,54]. Raschzok and colleagues [53] have demonstrated that labeled hepatocytes transplanted via intraportal infusion into the liver, via direct injection into the splenic parenchyma, or via intra-arterial infusion to the spleen of non-pretreated swine could be monitored *in vivo* as distinct areas of hypointensity following injection. However, injection of pure particles led to similar hypointense areas in the spleen, indicating that discrimination between labeled cells and free particles was not possible [53]. Following intraportal infusion, signal voids appeared in the liver and were histologically correlated to microthrombi of MPIO-labeled cells, which could not previously be detected by conventional imaging modalities. These findings contrast with the results from small animal models, emphasizing the need for large animal models [54]. Fast dynamic 'real-time' MRI yielding several images per second, has allowed for the noninvasive tracking of MPIO-labeled hepatocytes that were infused into the spleen of pigs [55]. However, relatively little was known about the effects of MPIO labeling on hepatocyte function.

Given the successful magnetic cell labeling achieved herein and as reported by others in other cell types, two challenges remain. First, MRI detects only the magnetic particle, independent of whether the particles are inside live or dead cells, or inside cells at all. Second, dilution of the magnetic particle to undetectable levels as cells divide makes it nearly impossible to detect large scale repopulation of the liver by transplanted cells. For example, “n” cell divisions results in 2^n^ dilution of agent. So, in the case of a cell containing 16 pg of iron, assuming equal distribution of magnetic material during cell division, after 5 cell divisions cells contain less than 1 pg of iron. While single MPIOs have been detected in vitro at high resolution [56], 1 pg iron per cells is a more practical limit of minimal detectability [57,58]. As such, MRI-based cell tracking may not be useful by itself in evaluating long term engraftment studies past the first few cell divisions [59]. For long term detection of hepatocytes transplanted ectopically, one could use a hepatocyte targeted agent to identify cells. Several *in vitro* and *in vivo* proof-of-principle studies have already demonstrated the feasibility of hepatocyte targeted agents [60–62]. For cells transplanted into the liver an MRI reporter system will likely be required for evaluating long term engraftment [63] and this will require the availability of a cell line that can be genetically modified *in vitro*, such as PICM-19FF. Until such a genetically modified cell is achieved and validated, the utility of MRI-based cell tracking of magnetically labeled hepatocytes may largely lie in evaluating the optimization of delivery routes and the efficiency of the initial seeding of the liver, still, two critical components for hepatocyte transplantation.
