# Correction: Magnetic Cell Labeling of Primary and Stem Cell-Derived Pig Hepatocytes for MRI-Based Cell Tracking of Hepatocyte Transplantation

**DOI:** 10.1371/journal.pone.0185524

**Published:** 2017-09-21

**Authors:** Dwayne R. Roach, Wesley M. Garrett, Glenn Welch, Dorela D. Shuboni-Mulligan, Thomas J. Caperna, Neil C. Talbot, Erik M. Shapiro

Dr. Dorela D. Shuboni-Mulligan is not included in the author byline. She should be listed as the fourth author and affiliated with 1: Molecular and Cellular Imaging Laboratory, Department of Radiology, Michigan State University, East Lansing, Michigan, United States of America. The contributions of this author are as follows: Conceived and designed the experiments, performed the experiments, wrote the paper.

The correct citation is: Roach DR, Garrett WM, Welch G, Shuboni-Mulligan DD, Caperna TJ, Talbot NC, et al. (2015) Magnetic Cell Labeling of Primary and Stem Cell-Derived Pig Hepatocytes for MRI-Based Cell Tracking of Hepatocyte Transplantation. PLoS ONE 10(4): e0123282. https://doi.org/10.1371/journal.pone.0123282
